# Probing Biosensing Interfaces With Single Molecule Localization Microscopy (SMLM)

**DOI:** 10.3389/fchem.2021.655324

**Published:** 2021-04-29

**Authors:** Xiaoyu Cheng, Wei Yin

**Affiliations:** ^1^State Key Laboratory for Modern Optical Instrumentations, National Engineering Research Center of Optical Instrumentation, College of Optical Science and Engineering, Zhejiang University, Hangzhou, China; ^2^Core Facilities, School of Medicine, Zhejiang University, Hangzhou, China

**Keywords:** biosensors, interfaces, single molecules, super-resolution imaging, fluorescence imaging

## Abstract

Far field single molecule localization microscopy (SMLM) has been established as a powerful tool to study biological structures with resolution far below the diffraction limit of conventional light microscopy. In recent years, the applications of SMLM have reached beyond traditional cellular imaging. Nanostructured interfaces are enriched with information that determines their function, playing key roles in applications such as chemical catalysis and biological sensing. SMLM enables detailed study of interfaces at an individual molecular level, allowing measurements of reaction kinetics, and detection of rare events not accessible to ensemble measurements. This paper provides an update to the progress made to the use of SMLM in characterizing nanostructured biointerfaces, focusing on practical aspects, recent advances, and emerging opportunities from an analytical chemistry perspective.

## Introduction

At the heart of measurement science lies the need to find answers to the following questions: 1. What is in the sample? 2. In what amount? The same questions are being asked repeatedly in applications including (but not limited to) drug discovery, environmental monitoring, cancer diagnostics, and SARS-COVID19 testing etc., all of which have broad social impacts on a global scale. However, asking questions is easy, finding answers to them are often hard, especially when the concentration of analytes is low and signals difficult to detect.

Single Molecule Localization Microscopy (SMLM) represents a class of fluorescent super-resolution imaging technique capable of detecting individual molecules with resolution at least a magnitude lower than the diffraction limit of conventional light microscopy. First demonstrated in 2006 and winning the Nobel Prize in Chemistry in 2014 (Dickson et al., 1997; Betzig et al., 2006; Rust et al., 2006; Moerner, 2015a,b), the elegant and robust design of SMLM allows direct observation of fluorescent molecules with resolution at molecular length scale using a standard bench-top microscope. The typical lateral resolution of SMLM is around 20 nm, and the axial resolution is below 100 nm (von Diezmann et al., 2017; Pan et al., 2018, 2019). Being a far field technique which can rely on large field-of-view detectors (Huang et al., 2011; Long et al., 2012; Ma et al., 2013), SMLM enables the probing of millions of single molecules simultaneously, which is hard to achieve with near-field scanning techniques. This has made SMLM an ideal tool for studying biosensing interfaces such that rare events can be detected and sample heterogeneity studied to enable building quantitative relationships inaccessible to ensemble studies (Titus and Willets, 2013a,b; Blythe et al., 2014; Nicovich et al., 2017; Pan et al., 2018; Cheng et al., 2019; Xing et al., 2019). Here, we provide a short account on the advances of using SMLM in the study of biosensors, focusing on application aspects, and recent progresses in the past several years.

## Basics of SMLM

**Figure 1:**
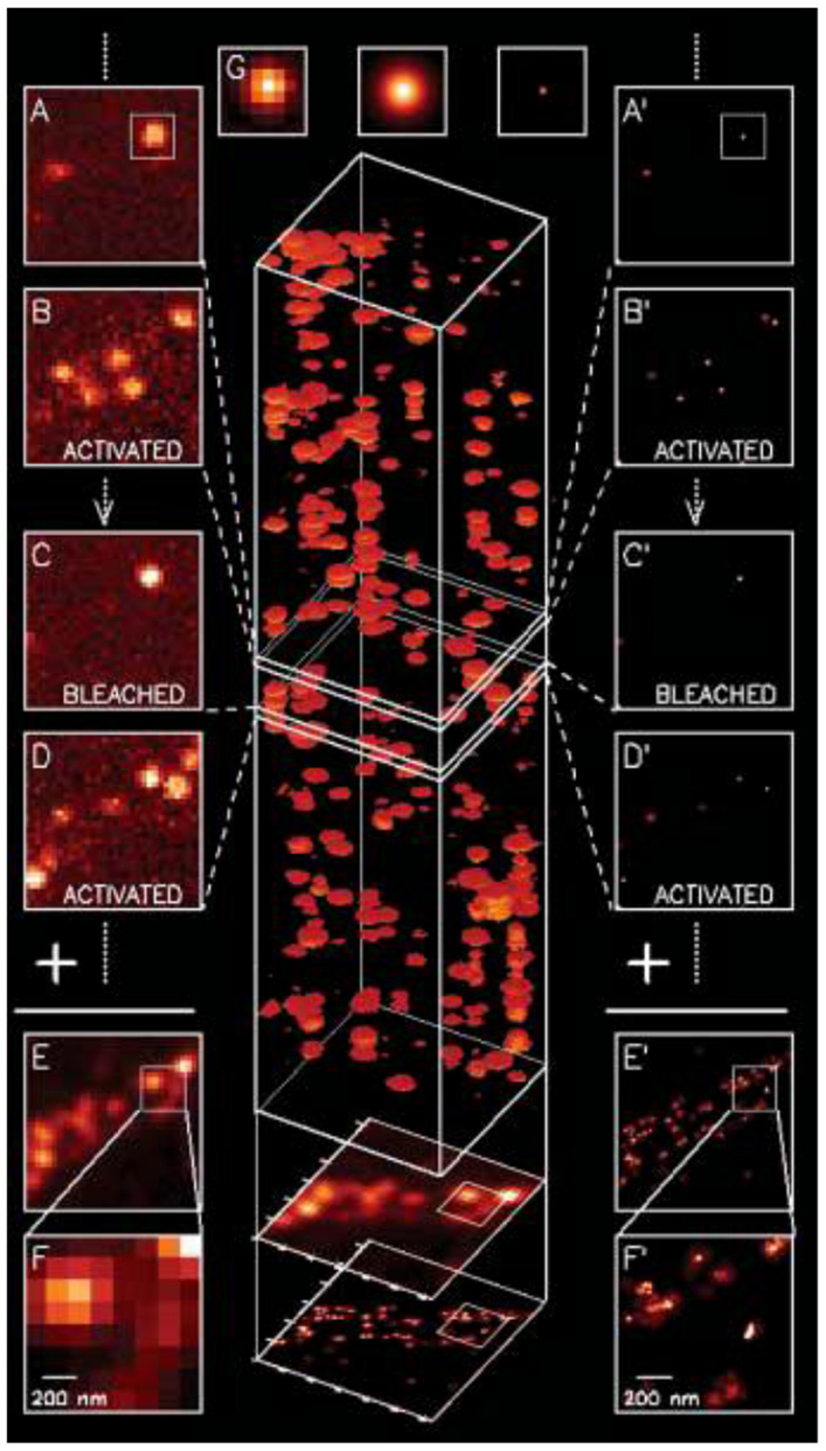
The first SMLM demonstrated with fluorescent proteins in 2006 (Betzig et al., 2006). A series of diffraction limited images were acquired, and to each image PSF for single emitters were localized with accuracy typically below ~20 nm and image reconstructed to form the final image. Copyright with permission from Science.

The foundations of SMLM have been reviewed thoroughly elsewhere, therefore they are only covered briefly due to the limited scope of this mini review (Moerner, 2015a,b). Fundamentally speaking, there are two building blocks to the mainstream SMLM techniques: scholastically photo-switching single molecules, and super-reconstruction. Briefly speaking, the process work via (1) accurate localization of point spread function (PSF) from single emitters, and (2) using various methods to allow only a limited number of emitters to be in the “ON” state so that (1) can be applied to each image plane to allow super-reconstruction, such as an early demonstration shown by Betzig et al. (2006) (Figure 1).

### Typical Methods to Induce Single Molecule Blinking

As a fluorescence technique, one of the requirements of SMLM is to observe probe molecules randomly switch between fluorescent “ON” and “OFF” states. After first observation of such phenomena in fluorescent proteins at room temperature in the late 1990s (Dickson et al., 1997), the past two decades have seen numerous SMLM probes developed and methods to blinking control (Li and Vaughan, 2018). Usually, fluorophores are more likely to stay in the fluorescent “ON” state. In practice, the challenge is often to find ways to extend the length of the “OFF,” or dark state, and to develop methods to reduce labeling density. Among the various approaches reported, three approaches have been established, and are the most frequently used in the today's mainstream SMLM.

**Figure 2:**
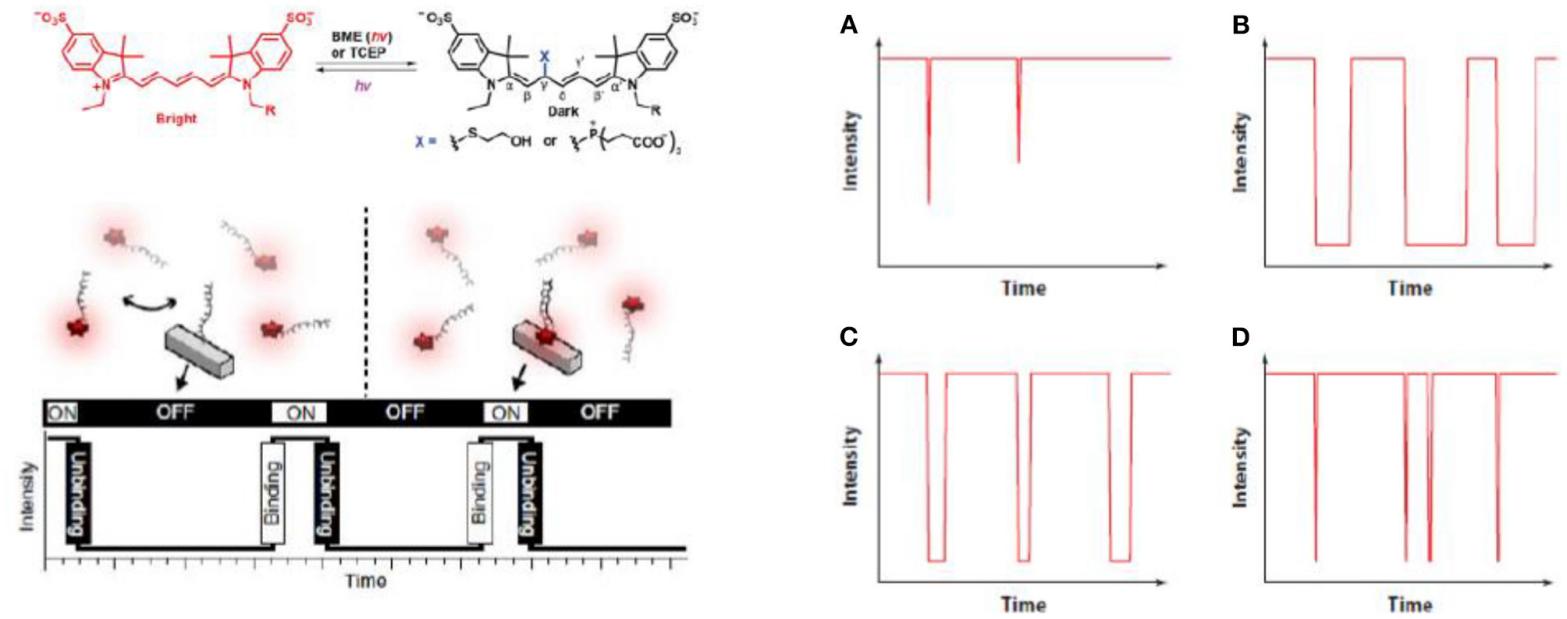
Three typical methods to introduce single molecule blinking. A carefully designed buffer environment extends the length of the “OFF” state (van de Linde et al., 2011; Sauer and Heilemann, 2017; Li and Vaughan, 2018) (Top). In PAINT, blinking control is achieved by transient adsorption of the imaging strand to the binding strand immobilized on the substrate (Jungmann et al., 2010; Stein et al., 2019) (Bottom). Triplet mediated blinking can be controlled in the gas phase without the use of solution environment once oxygen is removed and lifetime of triplet state extended (Ha and Tinnefeld, 2012; Blythe and Willets, 2016; Cheng et al., 2019) (Right). **(A)** Without oxygen removal. **(B)** Oxygen removed. **(C)** With an inefficient triplet quencher. **(D)** With an efficient triplet quencher. Copyright with permission from the American Chemical Society and Nature Publishing Group.

The first is direct photos-switching of immobilized fluorophores in a buffered environment (Nahidiazar et al., 2016) (Figure 2, top). This is the most commonly used approach in SMLM, with many examples demonstrated represented with dSTORM, STORM, PALM, etc. (van de Linde et al., 2011). A carefully designed buffer environment requires: (1) an oxygen-removing reaction, which typically involves glucose oxidation and this also reduces photo bleaching. (2) A reducing agent, such as β-mercaptoethanol or Tris(2-carboxyethyl)phosphine (TCEP), is often added to drive the fluorophores into the dark states (Vaughan et al., 2013), typically with the Alexa fluorophore family (e.g., Alexa 647). (3) Additional reagents, such as the enzyme horseradish peroxidase was used to reduce the amount of hydrogen peroxide, a radical generator. This is the most widely adopted approach to induce photo-switching for SMLM, and numerous studies have been reported in literature (Sauer and Heilemann, 2017).

The second group of methods is PAINT (Jungmann et al., 2014, 2016; Schnitzbauer et al., 2017; Stehr et al., 2019) (Figure 2, bottom). PAINT stands for points accumulation for imaging nanoscale topography. As a different method to the direct photo-switching methods mentioned above, PAINT does not rely on photo-switching of immobilized fluorophores in the imaging plane. Instead, fluorophores labeled imaging species, usually single stranded DNA, diffuse in the imaging buffer and bind transiently to the surface bound species to produce the fluorescent “ON” signal (Peterson et al., 2016b; Morris et al., 2018; Lackey et al., 2020). e.g., By controlling the length of the matching strand pairs, stochastic blinking of single molecular fluorophores can be achieved, and the lateral spatial resolution can be lowered down to ~5 nm (Dai et al., 2016). The very high resolution is possible because localization precision is related to the inverse square of the photon count, and in PAINT, high photon counts can be obtained when a longer stranded DNA is used, although to some extend the speed of imaging has to be compromised (Dai et al., 2016). A feature with PAINT is that multiple contact between probes and their target enables a higher total number of photons, hence higher resolution to the technique.

The third approach does not require a solution environment, but can be performed in the gas phase, as seen in Figure 2, right (Ha and Tinnefeld, 2012; Blythe and Willets, 2016; Cheng et al., 2019). In gas phase SMLM, fluorescent photons are observed as fluorophores cycling between singlet excited state and the ground state, with a small chance to enter the triplet state. The triplet state is non-emissive because electrons are parallel and spin-forbidden, the lifetime of which is significantly longer than the singlet state (“ON” state), e.g., millisecond to second. Hence, once a fluorophore enters the triplet state, it can remain in the dark for a long time, as long as oxygen is removed because oxygen is a triplet quencher (Blythe et al., 2015a,b; Blythe and Willets, 2016; Cheng et al., 2019). An advantage of triplet quenching induced SMLM is its good performance in an epi set-ups without buffer environment, which is hard to achieve with PAINT or dSTORM when the imaging strands freely diffuse, so typically objective based TIRF set-ups are used.

### Super Reconstruction: The Importance for Fiducial Correction

SMLM relies on stacking many images of blinking fluorophores into the same region of interest. Hence, the second building block to SMLM is super-reconstruction (Sage et al., 2015). Overall, the aim of super reconstruction is to develop methods to obtain images below the diffraction limit efficiently and accurately. Roughly speaking, this process involves three separate steps: the first is drift correction, the second single emitter localization and third image reconstruction. The past decade has seen rapid advances in our understanding of emission patterns of single emitters, namely point spread function (PSF), and now higher order PSF fittings are available to enable 3D super-resolution imaging (Hao et al., 2013; von Diezmann et al., 2017; Li et al., 2020). Higher order drift correction methods, such as 3D drift control, has been shown to work efficiently

Due to the limited length of this mini review, more commonly used methods for drift correction after imaging acquisition are not the focus of this article, which were reviewed thoroughly elsewhere (Mockl and Moerner, 2020).

**Figure 3:**
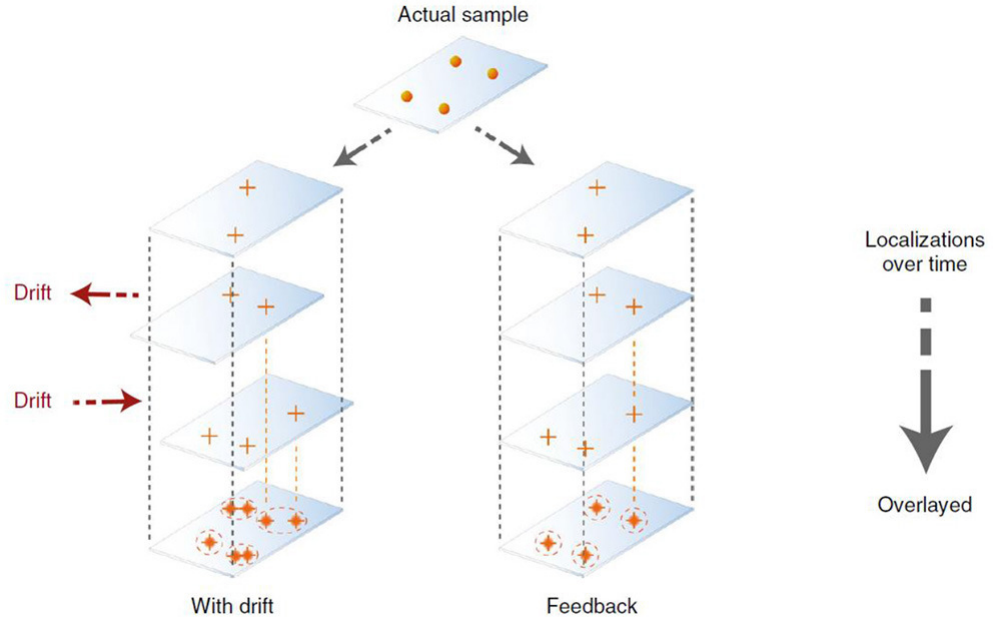
Active SMLM is achieved with better drift correction to improve the resolution without PSF engineering or change of fluorophores (Coelho et al., 2020, 2021). It was shown that using an actively stabilizing microscope, the lateral spatial drift could be reduced down to ~1 nm using self-calibrated piezostage and anti-shaking algorithms. This can be done without relying on complicated hardware and the entire process can be completed in 2 weeks. Copyright with permission from Nature Publishing Group.

Apart from PSF engineering, a recent development in super reconstruction is the realization of the importance of drift correction (Figure 3). In the past, attentions have been focused on PSF engineering so that advanced imaging concepts, e.g., high speed 3D imaging, can be done, in some cases close to video rate (Huang et al., 2013). Since images of SMLM were acquired over an extended period of time (e.g., *tens to hundreds of seconds*), the physical movement of the sample stage could induce significant aberrations. Solving this issue leads to SMLM microscopy with superior performance. In a recent study by Coelho et al. (2020), it was shown that actively stabilizing the microscope sample stage over the imaging process frame by frame could reduce the spatial drift down to ~1 nm (Coelho et al., 2020). Rapid repositioning of the samples using self-calibrated piezostage and anti-shaking algorithms were shown to reduce drift far below the photon-limited localization precision and improved the spatial resolution, and this does not require complex algorithms or hardware to achieve (Coelho et al., 2020). This is important because this technique can be applied without using a second laser beam, as seen in the single molecule depletion imaging via non-linear optical properties of the probes (Balzarotti et al., 2017).

## Application Toward Quantitative Bioanalysis

Initially established as an advanced imaging tool to probe biological structures (Tuson and Biteen, 2015), the applications of SMLM is advancing rapidly into the field of surface analysis including catalysis and biosensors (Sambur et al., 2016; Chen et al., 2017). Here we focus on the latter topic due to the very few reviews available to this topic at the moment, here three types of applications are addressed: PAINT for single molecule kinetic measurements; quantitative single molecule sensors with counting; and the emerging use of SMLM for predictive chromatography separations.

### PAINT: From Imaging to Kinetic Measurement

**Figure 4:**
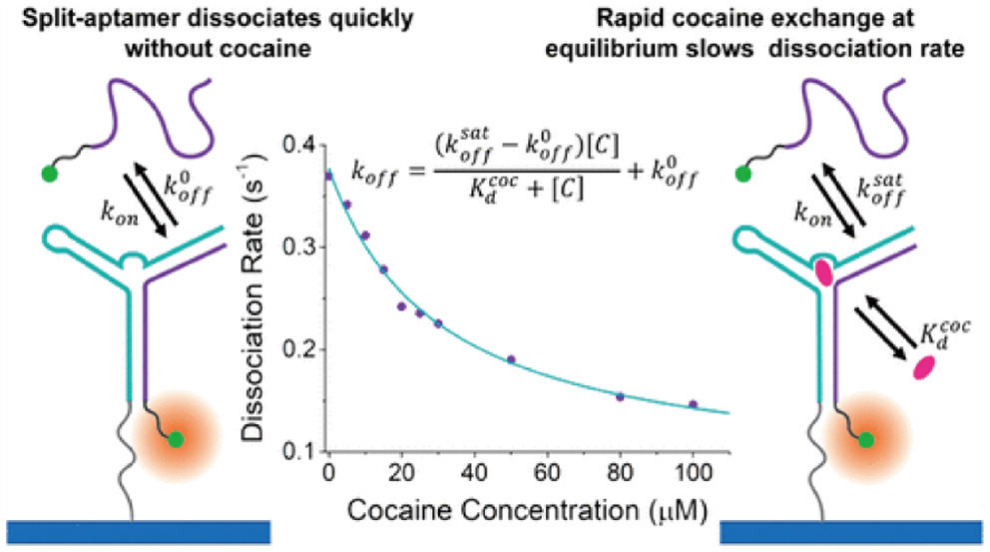
Measurement of single molecular binding kinetics with PAINT. Initially developed as an imaging tool, PAINT has been established as a method to measure binding affinities between biomolecules including DNA, peptides, and small molecular drug targets. Shown above as an example of measuring dissociation constant between cocaine and single stranded DNA split aptamer (Morris et al., 2018). Copyright with permission from the American Chemical Society.

As mentioned earlier, PAINT represents a subtype of SMLM techniques that relies on transient binding of biomolecules onto a surface immobilized molecule to induce photo-switching. Since the time each molecule resides on the imaging plane is related to the binding affinity between the molecular pairs at chemical equilibrium, important kinetic information, such as dissociation and/or association constant, namely *K*_*d*_ or *K*_a_, can be extracted by analyzing the fluorescent “ON” period in relation to the “OFF” period in the single molecular time trace (Jungmann et al., 2010). e.g., A series of studies by Harris's group showed measurement of molecular binding kinetics could be studied at single molecular level with PAINT, demonstrated by several examples with e.g., biotin-avidin, structure-switching aptamer, peptides, and small molecular targets etc. (Fox et al., 2009; Wayment and Harris, 2009; Heider et al., 2011; Peterson et al., 2016a; Morris et al., 2018; Peterson and Harris, 2018; Lackey et al., 2020) as seen in Figure 4.

The success of PAINT in studying molecular interactions at a single entity level is inspiring (Figure 4). PAINT is distinct to conventional methods for probing molecular interactions at biointerfaces, such as surface plasmon resonance sensors which typically relies on change of local dielectric environment therefore an ensemble measurement tool (Jiang et al., 2016, 2017). A clear advantage is that PAINT is capable of detecting rare events and sample heterogeneity, a distinct feature to single molecule tools. Saying this, due to the weak binding between single stranded DNAs, the robustness of DNA PAINT in response to the influences of environmental factors, such as salt concentration and temperature is yet to be explored, which could vary significantly in complex sensing environments, such as blood or serum. Another factor to consider is that PAINT remains as a fluorescent technique, but in many applications, the candidate molecule are non-fluorescent hence it is unlikely for PAINT to completely replace current methods in the near future.

### Counting Single Molecules on Biosensing Interfaces

Single molecule counting is another topic currently attracting broad attentions. From a quantitative biology perspective, knowing the exact number of molecules, especially proteins, in complex biological environments such as cells is of enormous importance and interest. This is because such knowledge will not only produce insights to how cells function and communicate, but could establish completely new ways of studying biological systems when information is gathered from single events instead of ensemble approaches currently being used (Zanacchi et al., 2017). In other words, single molecule counting allows SMLM to be treated as a bottom-up bioanalytical tool to generate discrete signal response, instead of simply an imaging tool to break the diffraction limit of light microscopy whose mechanism has been well-appreciated (Gooding and Gaus, 2016; Lu et al., 2017; Liu et al., 2018). This marks a conceptual recent advance of SMLM, and it is most relevant to the analytical science community.

Though it sounds attractive, counting the absolute number of single molecules is by no means a trivial task. This is especially true for cellular imaging, in which counting accuracy is impaired by difficulties in controlling labeling density of the probes or complex photophysics of the blinking fluorophores. e.g., Some probes can remain in the dark and never switch “ON,” others could all switch randomly at different rates. Fluorescence is a process reflecting electrons transferring between ground/excited states to emit photons. Electrons within different bands are fermions and follow rules of Boltzmann's distribution, photons are Boson particles in nature and follow Poisson's distribution, the complex origins governing the single molecular blinking imposes significant challenges to build mathematical relationships, even empirical ones.

For biosensors, challenges to the labeling issue can be partially resolved, because the density of surface epitopes can be controlled at the interface. From an analytical chemistry point of view, this is important because in principle quantification can be done directly without amplification of the analytes or accumulation of the signal. This is a distinct advantage of SMLM sensors to other single molecule assays such as digital polymerase chain reaction, or single molecule optical arrays (Wu et al., 2019).

**Figure 5:**
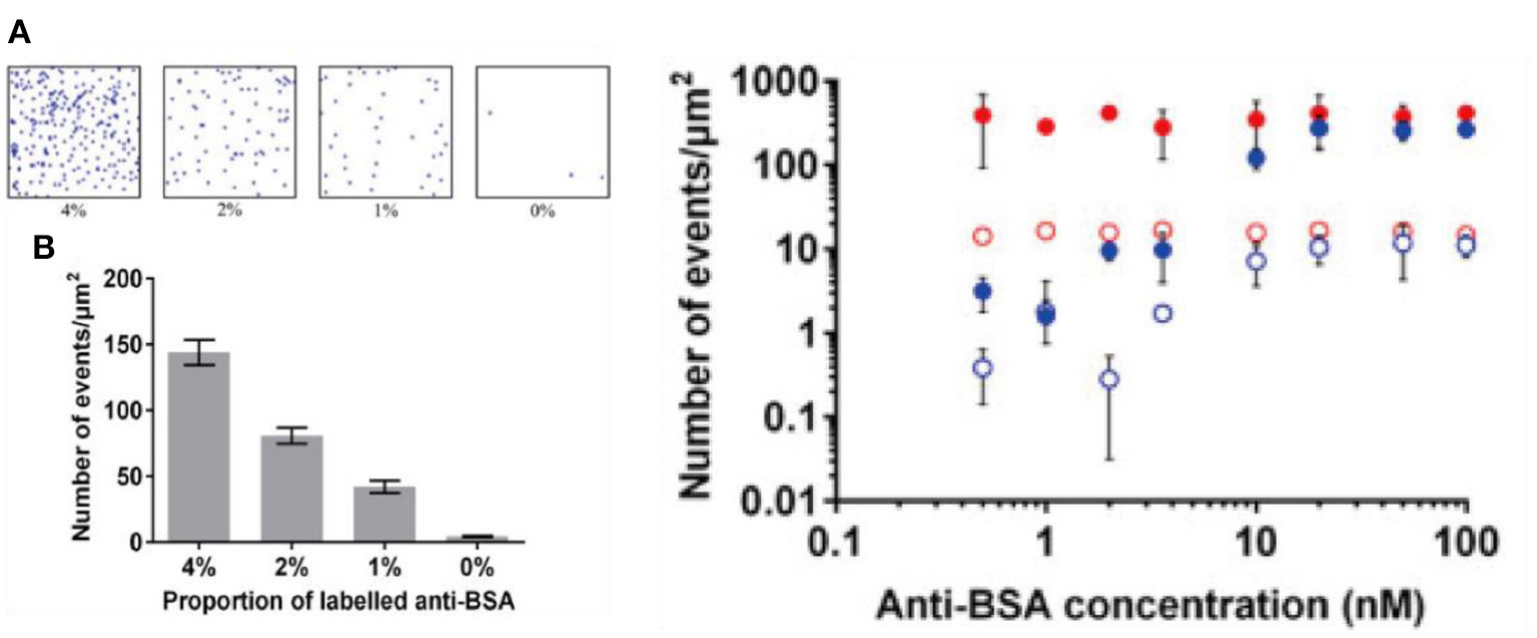
Toward immuno-biosensors with SMLM. Shown above: (Left) **(A)** raw images of bovine serum albumin proteins immobilized on glass coverslips with self-assembled monolayer on transparent conducting oxide substrate. **(B)** A quantitative relationship is built with increased amount of labeled proteins on the surface (Right) adding an increasing amount of antibody results in an increasing number of counting per image area (Lu et al., 2017). Copyright with permission from Elsevier.

One example was demonstrated by Lu et al. (2017). In a recent report, it was shown that images of SMLM could be obtained using fluorophore labeled antigens immobilized onto substrate modified by self-assembled monolayers with density of probes well-controlled (Figure 5) (Lu et al., 2017). An evident quantitative relationship was found when an increasing amount of antibodies was added. This study was important because it demonstrated the first proof-of-concept quantitative immuno-biosensors with SMLM. Though it sounds straightforward, absolute counting of single molecules on biosensing interfaces is not a trivial task. In practice, counting usually relies on preliminary knowledge of the blinking behavior of molecular targets, or calibration toward a reference (Jungmann et al., 2010). Common issues include: 1. A blinking fluorophore can enter the dark state due to various reasons including photo-bleaching, reacting with the reducing agent, or entering triplet state, each with a significantly different lifetimes. 2. A fraction of fluorophore may never turn “ON.” All of which can lead to over counting or under counting issues (Lee et al., 2012).

To date, absolute counting of single molecules with SMLM is still difficult to achieve, especially when biological samples are used when calibration can be difficult and target molecules tend to cluster together while the sample is heterogeneous. Additional challenges are associated with issues such as data interpretation and linking (Lee et al., 2017). Saying this, some successes have been achieved. e.g., In a recent study, combining SMLM to fluorescent correlation produces localization-based fluorescence correlation spectroscopy, or lbFCS (Stein et al., 2019). Essentially, the method might be the first to allow absolute counting of single molecules using SMLM, but due to the relatively complicated multimodal system it depends on, which is off-putting to non-experts, to what extent the technique can be widely used is still to be explored.

### Application to Analytical Bioseparation

Chromatography separation is a basic technique used in many industrial chemical production processes such as antibody drug developments. Bringing a new biopharmaceutical product into the market is both time consuming and cost ineffective, and during this process, nearly half of the effort is spent on the separation and purifications (Calabrase et al., 2020). Conventional analytical chromatography relies heavily on empirical trials and currently there is only very limited ability to predict their outcomes (Kisley and Landes, 2015). Due to the complexity of protein structures and significant sample heterogeneity (e.g., size, charge, structure), large amount of resources are being spent to optimize column separation efficiency. At the moment, methods to improve the performance of protein chromatography techniques are in urgent need.

**Figure 6:**
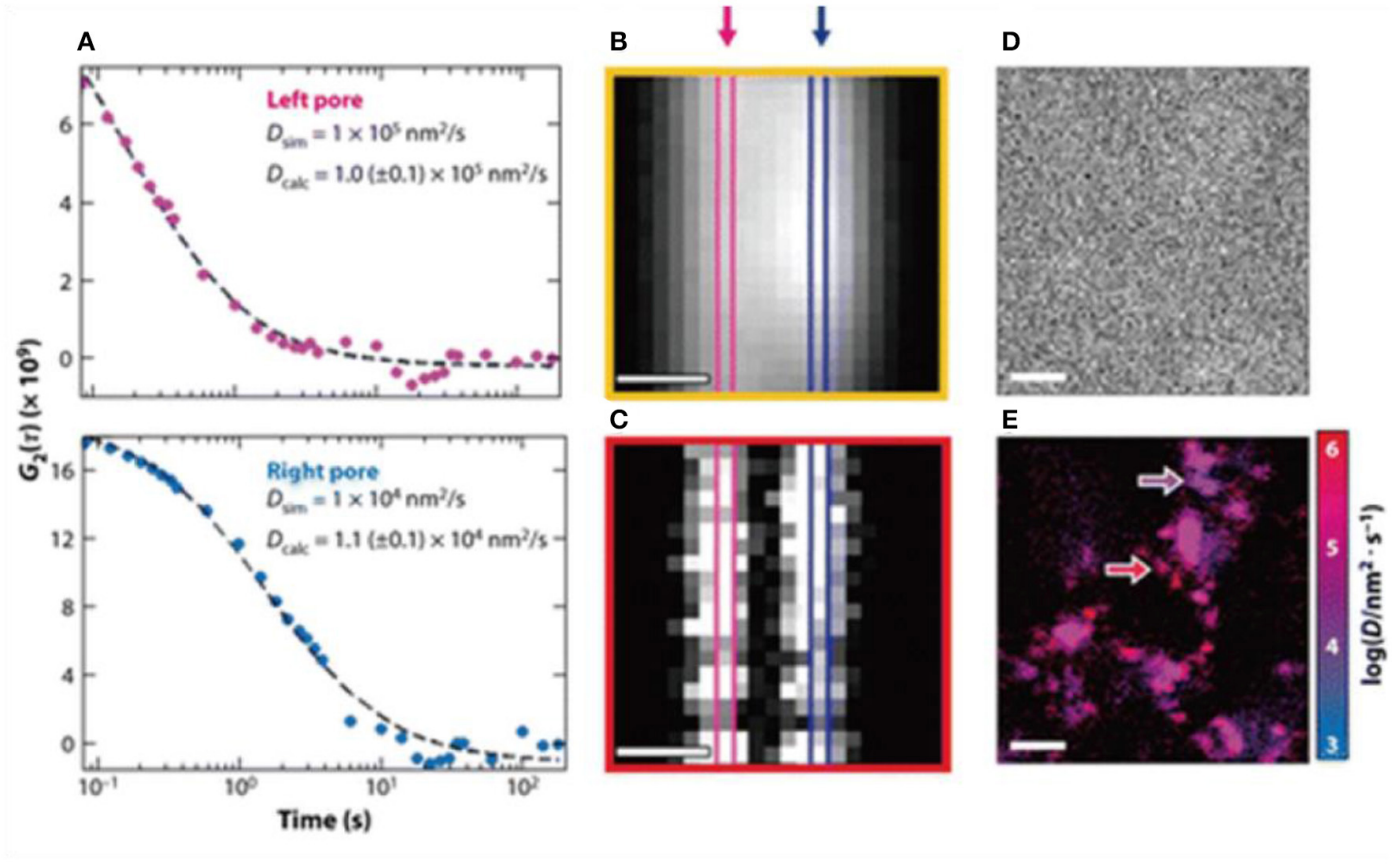
**(A–E)** Toward predictive protein chromatography via SMLM. It was shown that time resolved super-resolution imaging techniques and combining fluorescence correlation to fluctuation could help build new theoretical frameworks to expand the current understanding of the protein separations in column chromatography. Shown above as an example of using dual mode correlation spectroscopy and fluctuation imaging, so that heterogeneous diffusion behavior of proteins could be resolved and parameters derived (Moringo et al., 2018). Copyright with permission from American Chemical Society.

Single molecule optical methods may point to a possible direction to potentially solve this issue. In principle, SMLM allows quantification of protein-substrate interactions at a single molecular level. This has enabled improvements in the random theory of chromatography, with the aim of making it more quantitative and predictive (Figure 6) (Calabrase et al., 2020) e.g., in the recent works, it was shown that rapid imaging with time resolved single molecule detection, and combining fluorescence correlation spectroscopy and fluctuation imaging can be applied to the study of proteins diffusing through porous materials (Calabrase et al., 2020). Although strictly speaking this is still not an SMLM technique, it was shown that rare events that interfere with efficient protein separations could be identified and several quantitative relationships proposed by studying them. These developments were inspiring, because they represent a possible route for predictive chromatography single molecule technique design, hence a possible direction for applications of SMLM.

## Challenges and Emerging Directions

### Mass Transport and Target Enrichment

Mass transport is a fundamental process that describes the motion of analytes within their environments, which has been studied extensively in the field of electrochemistry and often the discussion is centered around Butler-Volmer equation. e.g., The focus is often centered on whether electron transfer or mass transport dominates the signal response (Wen et al., 2013). In the context of single molecular biosensors, mass transport is addressed by the insufficient interactions between the target molecule and sensing interface, especially when the analyte is present at low concentrations and Brownian motion inadequate. e.g., Studies with nanopores sensors suggested it could take over 10 min for the analyte to diffuse through the pore when the concentration is very low (Ying and Long, 2019) (e.g., <pM). In other words, even with a *sCMOS* with single molecular resolution, in practice the testing cycle could be hours, and therefore impractical (Wu et al., 2019).

**Figure 7:**
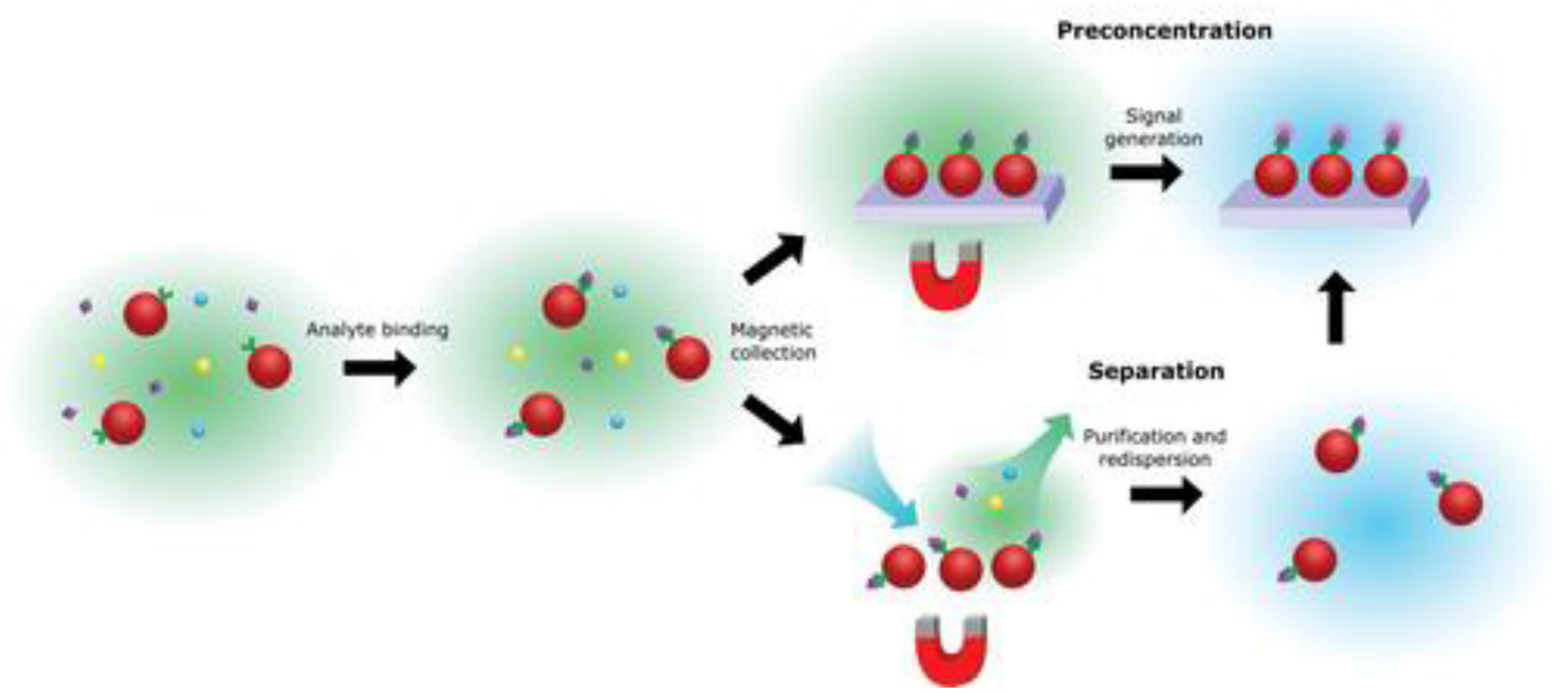
Basic principles of magnetic enrichment. Being a “hero” in solving the mass transport issue for ultrasensitive biosensors, the use of magnetic nanoparticles can be used for pre-concentration and/or separation of the analyte, while at the same time reducing non-specific effects (Gloag et al., 2019). Copyright with permission from Wiley.

One simple solution to this issue is magnetic enrichment, as shown in Figure 7. It has been shown that incorporating magnetic nanoparticles into the analysis could substantially reduce the limit of detection and also reduce non-specific effects via target enrichment (Tavallaie et al., 2018), as discussed in a recent review (Gloag et al., 2019). Currently, there are still limited studies on the use of magnetic nanoparticles for such uses in SMLM, so a clear opportunity exists. Saying this, caution is still needed, because magnetic nanoparticles are often coated with gold because of its well-established surface chemistry (Rosi and Mirkin, 2005). It is known that at this length scale, gold nanoparticles exhibit plasmonic effects, which are coherent oscillation of conducting *d* band electrons in response to the incident light (Willets et al., 2017). In the context of SMLM, plasmonic effects can potentially produce two outcomes, the first is that the emitting center can be shifted by the complex plasmon-molecular interactions (Xia et al., 2019), shrinking the images, and this is not favored (Cheng et al., 2019). The second is that it could produce significant signal enhancement which leads to higher localization precision, and is hence preferred. Hence, whether in this context, working with plasmonic metal is necessary or not is yet to be determined (Baiyasi et al., 2019).

### Non-specific vs. Specific Interactions

For biosensors to operate in complex environments, such as blood or serum, a key aspect to consider is non-specific interactions (Jiang et al., 2020). e.g., Nucleic acid analysis and immuno-biosensors typically rely on the specific binding of biomolecules on the sensing interface to induce an optical or electrical response. For such sensors, a non-specific effect is usually undesired and should be minimized. Typically, reducing non-specificity requires the use of a chemical antifouling layer or nanostructured interface, e.g., polyethylene glycol, zwitterionic layer, or mesoporous surfaces, etc. (del Rio et al., 2019; Jiang et al., 2020).

**Figure 8:**
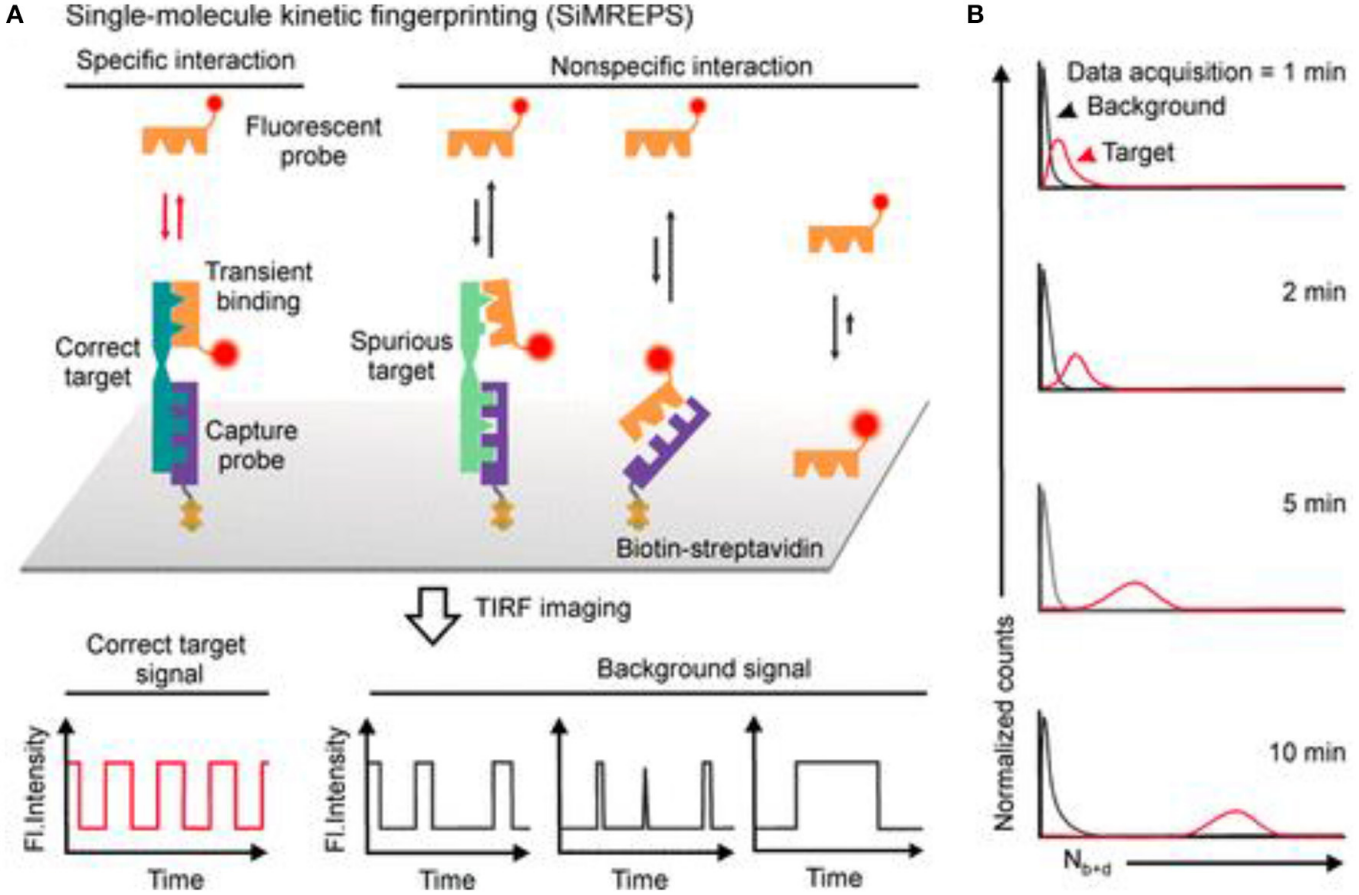
Using SMLM to differentiate specific vs. non-specific effects with PAINT. In SiMREPS, **(A)** using carefully designed molecular probes, specific interactions are associated with single molecule blinking signals that are homogeneous over time, while non-specific events are seen as heterogeneous blinking events. **(B)** Over time, better separation between specific and non-specific events are expected (Mandal et al., 2020). Copyright with permission from the American Chemical Society.

Recently, SMLM has been shown to be able to differentiate specific and non-specific interactions by exploring the single molecule time traces, as seen in Figure 8. e.g., In a recent development, an amplification-free approach, named single-molecule recognition through equilibrium Poisson sampling (SiMREPS) has been shown to be able to detect miRNA with LOD close to 1 fM (Johnson-Buck et al., 2015, 2019; Chatterjee et al., 2020; Li J. M. et al. et al., 2020). In SiMREPS, specific and non-specific interactions are resolved by inspecting different kinetic time traces at a single molecular level. e.g., Specific interactions produce homogeneous blinking events, while non-specific events generate single molecular signals that are more random and heterogeneous. This development is important, because it is one of the first examples of using SMLM to differentiate specific and non-specific binding, which is pivotal to nearly all biosensors.

## Conclusions

First demonstrated in 2006, it has been 15 years since the first demonstration of SMLM. Numerous methods of SMLM techniques have been developed, and now it is finding more applications in ultrasensitive bioanalysis. The realization of drift correction in SMLM is significant because it could potentially make sub-5 nm optical microscopy a routine technique without high-end engineering expertise and this is the length scale of a fluorescent protein. Kinetic measurement with PAINT, quantitative single molecule sensors and application to separation science are just three examples of emerging research frontiers brought about by SMLM. In the context of ultrasensitive sensing, to solve the mass transport issue for low abundance analyte detection, magnetic enrichment may present a viable solution, but caution is needed when a plasmonic system is used. Specificity is a fundamental issue to many bioanalytical devices operating in complex sensing environments, SMLM could lead to new knowledge to it and potentially offer a solution. Overall, the past years have witnessed rapid advances of using SMLM to probe biosensing interfaces. It is expected that the relevant field will continue to develop in an accelerated rate.

## Author Contributions

All authors listed have made a substantial, direct and intellectual contribution to the work, and approved it for publication.

## Conflict of Interest

The authors declare that the research was conducted in the absence of any commercial or financial relationships that could be construed as a potential conflict of interest.

## Abbreviations

GFP: Green Fluorescent Protein
dSTORM: Direct Stochastic Optical Reconstruction Microscopy
GSDIM: Ground State Depletion with Individual Molecular Return
PALM: Photo-activated Localization Microscopy
PAINT: Points Accumulation for Imaging Nanoscale Topography
SMLM: Single Molecule Localization Microscopy
STED: Stimulated Emission Depletion Microscopy
STORM: Stochastic Optical Reconstruction Microscopy
TIRF: Total Internal Reflection Fluorescence.
